# Revealing the burden of maternal mortality: a probabilistic model for determining pregnancy-related causes of death from verbal autopsies

**DOI:** 10.1186/1478-7954-5-1

**Published:** 2007-02-08

**Authors:** Edward Fottrell, Peter Byass, Thomas W Ouedraogo, Cecile Tamini, Adjima Gbangou, Issiaka Sombié, Ulf Högberg, Karen H Witten, Sohinee Bhattacharya, Teklay Desta, Sylvia Deganus, Janet Tornui, Ann E Fitzmaurice, Nicolas Meda, Wendy J Graham

**Affiliations:** 1 Immpact, University of Aberdeen, Aberdeen, Scotland, UK; 2 Immpact, Centre Muraz, Bobo-Dioulasso, Burkina Faso; 3 Centre de Recherche en Santé de Nouna, Nouna, Burkina Faso; 4 Department of Obstetrics and Gynaecology, Umeå University, Umeå, Sweden; 5 Tigray Regional Health Bureau, Mekelle, Ethiopia; 6 Immpact, Tema Hospital, Ghana; 7 Immpact, Noguchi Memorial Institute for Medical Research, Accra, Ghana

## Abstract

**Background:**

Substantial reductions in maternal mortality are called for in Millennium Development Goal 5 (MDG-5), thus assuming that maternal mortality is measurable. A key difficulty is attributing causes of death for the many women who die unaided in developing countries. Verbal autopsy (VA) can elicit circumstances of death, but data need to be interpreted reliably and consistently to serve as global indicators. Recent developments in probabilistic modelling of VA interpretation are adapted and assessed here for the specific circumstances of pregnancy-related death.

**Methods:**

A preliminary version of the InterVA-M probabilistic VA interpretation model was developed and refined with adult female VA data from several sources, and then assessed against 258 additional VA interviews from Burkina Faso. Likely causes of death produced by the model were compared with causes previously determined by local physicians. Distinction was made between free-text and closed-question data in the VA interviews, to assess the added value of free-text material on the model's output.

**Results:**

Following rationalisation between the model and physician interpretations, cause-specific mortality fractions were broadly similar. Case-by-case agreement between the model and any of the reviewing physicians reached approximately 60%, rising to approximately 80% when cases with a discrepancy were reviewed by an additional physician. Cardiovascular disease and malaria showed the largest differences between the methods, and the attribution of infections related to pregnancy also varied. The model estimated 30% of deaths to be pregnancy-related, of which half were due to direct causes. Data derived from free-text made no appreciable difference.

**Conclusion:**

InterVA-M represents a potentially valuable new tool for measuring maternal mortality in an efficient, consistent and standardised way. Further development, refinement and validation are planned. It could become a routine tool in research and service settings where levels and changes in pregnancy-related deaths need to be measured, for example in assessing progress towards MDG-5.

## Background

The Fifth Millennium Development Goal (MDG-5) calls for a 75% reduction in maternal mortality by 2015. Measuring this target with sufficient precision to show such a downward trend is, however, a major challenge, particularly in high mortality settings with weak health information systems[1,2]. Deaths for more than 50% of the world's population go unrecorded in official statistics, including the majority of pregnancy-related deaths[3]. Thus developing practical methods which can consistently and reliably help identify otherwise missed pregnancy-related deaths in the community is an urgent priority in order to show and inform progress towards MDG-5.

Verbal autopsy (VA) is an established method of ascertaining likely causes of death in settings where death registration is non-existent or inadequate. Relatives or caretakers of the deceased are interviewed to elicit the circumstances and symptoms of the death. These data are then interpreted to derive likely causes of death; however, it is at this stage that the limitations of VA become most apparent. Commonly, local physicians assign individual causes of death based on information from the VA interview. This takes time and consumes valuable skilled resources, often in settings where physicians are scarce. Moreover, the reliability and repeatability of interpretation by physicians has been questioned [4-9]. In Bangladesh, for example, one physician attributed 41% of all maternal deaths to direct obstetric causes, while another group determined the proportion as 51%[5]. Such discrepancies can be misleading and preclude comparisons of cause-specific mortality determined by physician review between regions and over time, where different physicians and their methods of interpreting evidence may differ. How to accurately interpret VA interview material, especially for a large number of cases, thus remains a major methodological obstacle. Approaches such as algorithms and neural networks have been tried inconclusively in some settings, and have often been criticised for excluding multiple causes of death[4,7].

Monitoring progress towards MDG-5 requires population-based identification of deaths among women of reproductive age (occurring in health facilities, at home, or elsewhere), followed by determination of cause in order to identify maternal deaths[10]. Methodological limitations in interpreting VA data are therefore a significant barrier to accurately measuring the burden and causes of pregnancy-related death and preclude rigorous evaluations of the effectiveness of safe-motherhood intervention strategies. In order to tackle the problem of maternal mortality, and move closer to achieving MDG-5, new approaches to measuring cause-specific mortality among women of reproductive age need to be developed, validated and discussed.

## The InterVA model[11]

Recent development of a probabilistic approach to VA interpretation designed to overcome the weaknesses of physician reviews and algorithms has delivered promising results [12-14]. Based on Bayes' theorem, this novel approach processes indicators from VA data rapidly and lists up to three likely causes of death for each case. A likelihood for each cause of death and an overall certainty factor is reported. Validation of the InterVA model in Vietnam and Ethiopia, settings with markedly different mortality patterns, correlated well with local physician reviews, and the one model was able to reflect local variations in mortality. Detailed descriptions of the development of the probabilistic model and its validation are available elsewhere [12-14].

This paper describes the adaptation of the general InterVA model into a specialised maternal version, InterVA-M, for interpreting VA data for deaths of women of reproductive age (15–49 years), and compares findings with previous physician reviews. A more complex and specialised model was necessary for pregnancy-related deaths, to include assessment of a particular death being pregnancy-related, as well as more detailed pregnancy-related causes of death and corresponding indicators.

## Methods

Based on previous work, a list of signs, symptoms and causes of death for women of reproductive age was agreed upon by an experienced international physician panel, familiar with clinical practice in developing countries and the process of VA. Probabilities reflecting the occurrence of each cause and each indicator among female deaths in the 15–49 year age range, and for each indicator given a specific cause, were determined by the panel using a Delphi technique. The InterVA-M model then uses a Bayesian approach to process these probabilities, implemented using Microsoft Visual FoxPro. As with the all-cause InterVA program, InterVA-M presents up to 3 causes of death, each with a percentage likelihood, and an overall certainty factor, defined as the average of the likelihoods. Cases with more detailed VA data usually result in higher likelihoods and hence a higher overall certainty. In addition, the maternal model attributes a likelihood for each case being pregnant at death, dying within 6 weeks of pregnancy ending, or not being recently pregnant.

The InterVA-M model was tested and assessed in collaboration with the Centre de Recherche en Santé de Nouna (CRSN), a demographic surveillance site (DSS) situated in rural north-west Burkina Faso. CRSN has undertaken VA routinely over a long period, in an area typical of the high maternal mortality ratio found in Burkina Faso (1,000/100,000 live births) [15-17].

Since 1992, CRSN fieldworkers have followed up every death, after a culturally appropriate period of three months' mourning, by interviewing a friend or family member using a VA questionnaire which has evolved in close collaboration with WHO and INDEPTH [18] and been adapted for the local setting. Wherever possible, the primary caretaker of the deceased immediately before death was interviewed and information relating to the signs, symptoms and circumstances of death collected. The questionnaire consisted of open-ended as well as fixed-response questions.

Completed VA forms for all deaths in the Nouna DSS since 1992 were scrutinised and those pertaining to females aged 15–49 years extracted and recorded in a spreadsheet format. This yielded data on 380 deaths, each of which had been assessed by local physicians. Approximately one third of these cases (n = 122) were used for initial testing of the probabilistic model to highlight any errors or omissions, which were then presented to and discussed by the expert panel. In addition to the 122 cases from CRSN, archived VA data for 203 obstetric-related deaths from Bangladesh, 18 adult female deaths from Ethiopia and 15 adult female deaths from Ghana were also used in this initial testing to avoid modelling the Burkina Faso setting too specifically. From this process, a definitive list of 80 indicators and 21 causes was formulated as the basis of the model for further assessment, as shown in Table 1.

**Table 1:** Indicators and causes of death used in the InterVA-M model

Indicators		Causes
was she pregnant at death	had professional assistance at delivery	HIV/AIDS related death
died within 6 w of delivering a baby	was delivery by Caesarean	malaria
died within 6 w of early pregnancy ending	was delivery by forceps/Ventouse	tuberculosis (pulmonary)
said to be non-pregnant 6 wks before death	did uterus came out after delivery	hepatitis
was she married at time of death	any swelling of feet and ankles	cardiovascular disease
was death during wet season	any swelling of face	respiratory disease
was she aged under 20 yrs	any blurred vision	injury
was she aged 20 to 34 yrs	any acute abdominal pain	suicide
was she aged 35 to 49 yrs	any foul smelling vaginal discharge	cancer
had she ever been pregnant	any previous Caesarian section	---------------------------------------
was she breast-feeding before death	any stiff neck	not pregnant within 6 weeks of death
was the pregnancy unwanted	any excessive night sweats	pregnancy ended within 6 weeks of death
any attempt to terminate pregnancy	any enlarged swollen glands	pregnant at death
was this her first pregnancy	any persistent cough > 3 wks	---------------------------------------
were there >4 previous pregnancies	any persistent fever > 3 wks	haemorrhage
was this a multiple pregnancy	was she coughing up blood	pregnancy-related sepsis
was she < 3 months pregnant at death	any jaundice or yellowness of skin/eyes	non-pregnancy related infection
any history of acute fever	any ulceration or infected wound	obstructed labour
any required IV or IM antibiotics	was she immunized against tetanus	ruptured uterus
was there coma >24 hrs before death	did she require iron injections	pregnancy-induced hypertension
did she ever have fits	any diagnosis of epilepsy	abortion
any pallor and/or anaemia	any diagnosis of TB	anaemia
any general swelling of body	any diagnosis of HIV/AIDS	ectopic pregnancy
breathless carrying out normal activities	any diagnosis of thrush	
any weight loss	any diagnosis of Karposi's sarcoma	
Recently bed bound for most of day	any diagnosis of malaria	
any sudden collapse	any diagnosis of liver disease	
any blood transfusion required	any diagnosis of haemoglobinopathy	
hysterectomy shortly before death	any surgery in month before death	
any recurrent fever	any diagnosis of cancer	
any shivering with fever	any diagnosis of heart disease	
major bleeding in 1^st ^3 months of preg	any suggestion of recent injury	
major bleeding in pregnancy or delivery	any suggestion of suicide	
did the placenta remain inside		
was death within 24 hrs of preg ending		
any delay in reduction of uterus size		
was blood pressure raised during preg		
any proteinuria reported		
were fits only pregnancy related		
was labour prolonged >24 hr		
was a baby delivered alive		
did she die in labour undelivered		
was baby's position abnormal		
was baby too big for delivery		
was part of baby prolapsed		
was delivery at home		
was delivery at a health facility		

This probabilistic model was then assessed using the hitherto untouched 258 cases of adult female death from CRSN, by comparing the results from the probabilistic model with the original physician reviews. Following CRSN's usual procedures, initially two specially trained local physicians had reviewed each case, trying to reach consensus on a single cause of death based on adapted ICD-10 categories. Where no consensus was reached, a third physician, not blinded to the opinions of the other physicians, reviewed the data in order to arrive at a consensus cause of death.

Consensus was reached on a single cause of death by the two reviewing physicians in only 102/258 (39.5%) cases. The remaining 156 cases (60.5%) were reviewed by the third independent physician, after which a two-thirds majority consensus on a single cause of death was reached in a total of 240 cases (93%), although in 30 of these the result was "indeterminate". No consensus was reached in 18 cases (7%).

Given the diversity of the original physician opinions, for the purposes of this assessment each individual physician diagnosis was used, weighted according to the number of physicians reviewing the case. This allowed comparison with the InterVA-M model's possible multiple causes, which were weighted by their likelihoods. Thus the same original VA questionnaire data were processed independently by the original physicians and the model, giving individually assigned cause(s) of death by both methods. These were then aggregated to cause specific mortality fractions (CSMF) at the community level.

Emphasis is often placed on the importance of open-ended, free-text information collected using VA questionnaires[19,20]. This frequently includes verbatim accounts from respondents, which probably fit better with physicians' customary approaches to diagnosis than a series of closed questions. The omission of open-ended information from most algorithmic approaches to VA interpretation has been a major criticism. Thus indicators which were extracted solely from the free-text sections of the CRSN VA forms were distinguished, to assess the added value of the free-text information. All the cases were processed with and without including the exclusively free-text indicators.

This study received ethical approval from the Centre Muraz Ethical Clearance Committee, Burkina Faso and from the Institutional Review Board of CRSN. In addition, informed consent was obtained from participants before VA interviews were conducted.

## Results

**Figure 1:**
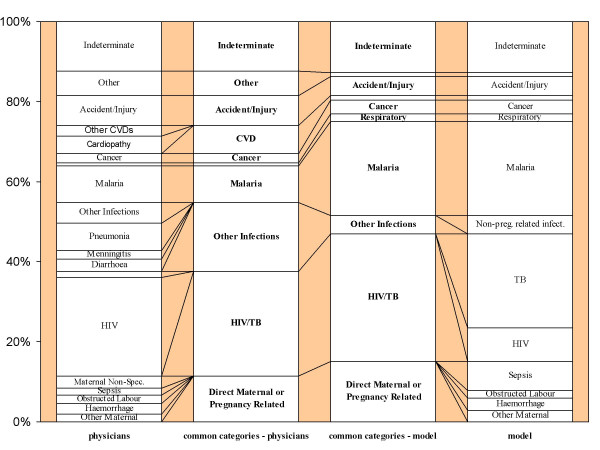
Representation of the burden of each of the major cause of death categories derived by local physician review and InterVA-M interpretation of VA data for 258 adult female deaths in Nouna, Burkina Faso. The two central columns represent groups of causes to facilitate comparison.

Examination of the range of causes assigned by physicians and InterVA-M revealed some important differences in terminology and cause of death categories. In many cases the physicians tended towards specific descriptions, for example "cardiopathy", in preference to more general cause of death categories, such as "cardiovascular disease". The following comparison is based on a rationalisation between the physician-assigned causes and the possible causes in the InterVA-M program. Figure 1 shows this, whilst also indicating the overall burden of each cause of death category in Nouna according to the two VA interpretation methods.

It was decided to group HIV/AIDS related deaths and deaths due to pulmonary tuberculosis (TB) into a single category for the assessment in this study since there was a large overlap of cases identified as being HIV/AIDS related by physician review and cases identified as being due to TB by the probabilistic model. Grouping HIV/AIDS and TB is reasonable given the significant clinical overlap between the two conditions and that around one quarter of those living with HIV are coinfected with TB[21].

**Figure 2:**
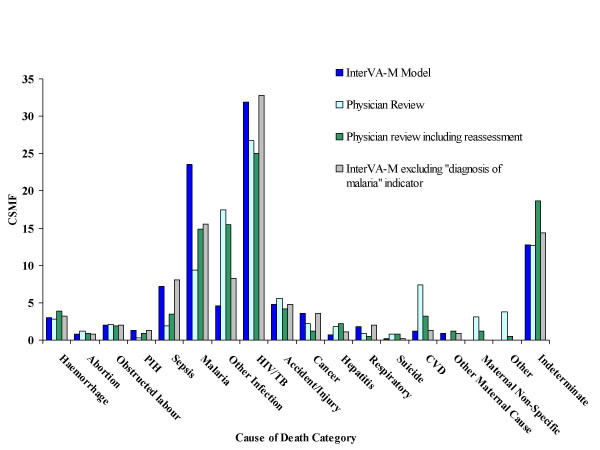
Cause-specific mortality fractions (CSMF) for 258 female deaths (15–49 years) from Burkina Faso according to different interpretations of verbal autopsy data.

Table 2 describes the case-by-case correlations between InterVA-M and physician reviews. In 123 cases (47.7%) the most likely cause as determined by the probabilistic model corresponds with at least one of the reviewing physicians. In an additional 25 cases (9.9%), at least one of the three most likely causes determined by the probabilistic model corresponds with at least one of the causes given by the physicians. In 110 cases (42.6%), the model's output contradicted the opinions of the reviewing physicians. However, in a number of cases, it was not clear that the physicians' diagnoses were more consistent with the VA data than those of the model. Therefore data from the 110 non-matching cases were presented to an additional, independent obstetrician, familiar with VA and obstetric practice in sub-Saharan Africa, who had not been involved in the development of InterVA-M (UH). This reassessment gave causes agreeing with the model in 63/110 cases (57.3%) (56 of which agreed with the model's most likely cause); causes which were in accordance with at least one of the original reviewing physicians in 18 cases (16.4%), and completely different causes for 29 cases (26.4%). Thus the model can be considered to have reached similar conclusions to a combination of the original physician reviews plus the physician reassessment in 211 (81.8%) of the overall cases. The CSMFs according to InterVA-M, original physician review and after reassessment are shown in Figure 2.

**Table 2:** Summary of case-by-case agreement between InterVA-M and physician review.

	correspondence of most likely cause	correspondence of any cause
original physician review	123 (47.7%)	148 (57.4%)
indicators from free-text removed	124 (48.1%)	147 (57.0%)
"malaria diagnosis" indicator removed	132 (51.2%)	154 (59.7%)
following physician reassessment	179 (69.4%)	211 (81.8%)

The model overestimated pregnancy-related sepsis compared with physician reviews, and underestimated other, non-pregnancy related infections and HIV-related deaths. Most contradictory cases identified as pregnancy-related sepsis by the model corresponded with physician diagnoses of non-pregnancy related infection and HIV-related death. However, since relatively few adult women die from infections in general, and in Burkina Faso approximately one third of deaths of females of reproductive age occur in pregnancy or within 6 weeks of pregnancy ending[22], then the prevalence of pregnancy-related sepsis compared with non-pregnancy related infections as determined by InterVA-M may be reasonable. The fact that pregnancy-specific indicators were included in almost half of these mismatched cases raises questions as to how the reviewing physicians originally interpreted this information.

The CSMF for cardiovascular diseases (CVD) varied between the two methods of interpretation, with physicians identifying more than six times the number of CVD deaths than the model. One third of the mismatched CVD cases, which included physician diagnoses of cardiopathy, hypertension, stroke and "*maux de coeur"*, corresponded to diagnoses of indeterminate cause by the model and most lacked any obvious CVD signs or symptoms, thus raising the question whether these diagnoses were simply describing the inevitable heart failure ultimately associated with death. The fact that physician reassessment identified the majority of these cases as indeterminate supports this, and suggests this may be a more appropriate public health outcome.

Malaria as a cause of death posed further diagnostic difficulties. Pregnant women are known to be at greater risk from malaria, yet malaria in pregnancy is frequently under-estimated, both clinically and in public health. Even in areas of relatively low transmission, many pregnant women may be infected at least once[23]. In Nouna, malaria is holoendemic, so most women will have been exposed to malaria before pregnancy and will have acquired some immunity. Nevertheless, semi-immune women are more susceptible during pregnancy with a clinical picture ranging from asymptomatic infection to severe, life-threatening illness[23,24].

Previous work on mortality in Nouna explained that malaria was not easily distinguished from other febrile illnesses using VA and the authors decided to group these causes together for the purposes of analysis[17]. Local physicians' tendency to diagnose other infections and febrile illnesses rather than malaria may be reflected in their diagnosis of approximately 30% of the mismatched malaria cases as non-pregnancy related infections, and a further 30% as HIV-related deaths. Since HIV-infected pregnant women are more likely to develop clinical malaria, it seems that there may be some overlap between these diagnoses that requires further consideration in refining the probabilistic model.

A further difference in malaria rates between the two methods was due to a history of malaria treatment in the VA interview being taken in the model as a "diagnosis of malaria". Although malaria treatment was commonly reported, it did not seem to greatly influence the reviewing physicians as to the cause of death, possibly reflecting their local knowledge of over-prescribing of antimalarials. To test this hypothesis, the model was re-run with all "diagnosis of malaria" indicators removed. This made the malaria CSMF for the two approaches more comparable (Figure 2), although the case-by-case agreement between InterVA-M and physician review was not significantly altered (Table 2).

The model assessed 21 women (8.1%) to have been pregnant at death (mean of individual likelihood values for pregnant at death = 79.4%), 56 (21.7%) to have died within 6 weeks of pregnancy ending (mean of individual likelihood values for death within 6 weeks of pregnancy ending = 85.4%), and 181 (70.2%) not to have been pregnant within 6 weeks of death (mean of individual likelihood values for not pregnant within 6 weeks of death = 87.8%). Thus pregnancy-related deaths amounted to 29.8% of mortality among women of reproductive age, of which half (15.1%) were attributed to direct maternal causes.

The effect of including or excluding exclusively free-text indicators in the input to the InterVA-M model made no significant difference to the results. Differences in agreement with physician opinions and in the magnitude of CSMFs were in all cases less than 1%.

## Discussion

Adapting the all-cause model for interpreting VA material from reproductive age female deaths gave CSMFs that were broadly comparable to physician reviews, in the absence of any available "gold standard", while also offering inherent consistency of interpretation over time and place. Reliable and consistent estimates of CSMFs, including attribution of mortality to pregnancy-related causes, at the population level, are the key requirements for monitoring MDG-5. Whilst statistical modelling may not reflect all the subjective subtleties that reviewing physicians might apply to individual cases, it offers very significant advantages in terms of efficiency, consistency and standardisation.

Rigorous validation of VA procedures is needed to establish confidence in the data collected, in order to understand the operational characteristics of VA in the populations under study and to identify misclassification patterns, which may then be corrected[25,26]. Poor validity for specific causes or cause-of-death categories raises questions not only about the utility of the specific VA tool, but also about the questionnaire used to collect data, interviewer skills and household awareness of health and disease. The extent of differences within the original physician diagnoses and the differences revealed in subsequent physician reassessment in the current study highlight the lack of standardisation inherent in physician interpretation of VA material. It was not possible, with these data, to robustly validate the model by comparison with original physician assessments. We accept that the process of reassessment by a further physician, as described above, may do more to illustrate the vagaries of VA interpretation than to provide a standard for validation, but it is important to recognise that in many settings there is no absolute "gold standard" by which to validate the performance of alternative VA interpretative models.

What is often termed "validation of VA" includes multiple components (validity and standardisation of VA instruments and interview, validity of VA interpretation(s), validity of arbitration between various interpreters and multiple validity issues around candidate "gold standards" such as medical record assessments). Discussions of VA validity typically focus on sensitivity, specificity and positive predictive values (PPVs) derived by comparing VA diagnoses with a reference diagnosis. In general, two types of reference "gold-standards" are used for validating VA tools: health-facility-based diagnoses or diagnoses derived from medical records, and community-based physician review diagnoses[7,19,27]. Whilst facility-based validations enable comparison of VA findings with a comparatively highly accurate medical diagnosis of cause of death, such studies are subject to selection and information bias and do not represent the populations for whom VA is intended, most of whom die without medical attention. Deaths from haemorrhage, for example, occur more rapidly than deaths from obstructed labour or pregnancy-related sepsis, and therefore they are likely to be under-represented in facility-based validations since haemorrhaging individuals will be less likely to reach a hospital before death; this is particularly true in areas with poor transportation.

Ideally therefore, the validity of VA should be assessed using a sample of community-based deaths. Physician review of VA data from community-based deaths has specific limitations, which have already been highlighted here and by others[9,19]. Issues of sampling communities for VA validation studies and the difficulty of tracing medical records (if they exist) to support physician diagnoses are further limitations of community-based studies.

Discussions of validity in terms of sensitivity, specificity and PPV assume that the referent diagnosis gives the right answer. This is reasonable if the objective is to assess whether alternate interpretation methods can be as accurate as the reference standards in the specific setting and time period of interest. It has been acknowledged, however, that VA diagnoses may be more accurate than the referent diagnosis in some instances[4,7,28]. Though not a formal validation of InterVA-M against the usual gold standards employed in VA studies, the current study compares the results from the probabilistic model with physician review of the data, and assesses the model's performance in terms of comparability, reliability and adequacy of purpose, avoiding reference to sensitivity, specificity or PPVs, which would imply inherent superiority of the physician review method. Efforts have been made to adjust imperfect gold standards, including adjusting for the quality and quantity of evidence in support of the reference standard[8,28] and such techniques may provide an opportunity for more thorough demonstrations of validity of InterVA-M against proxy gold-standards, at least for certain causes of death, in the future.

The proportion of deaths identified by the probabilistic model as being during pregnancy or within 6 weeks of pregnancy ending (30%) was slightly lower than the proportion of maternal deaths among deaths of all females of reproductive age for Burkina Faso as estimated by WHO, UNICEF and UNFPA (37%)[22]. However, this discrepancy is not simple to account for in the light of the modelling used to arrive at national estimates and the possibility of local differences in mortality patterns.

Categorisation of pregnancy-related deaths as direct or indirect, or pre-, inter- or post-partum, will never be easy using VA data. The current version of the model does not attempt an "intra-partum" category, although the combination of the pregnant/recently delivered categorisation with specific maternal causes can reveal this to some extent. In principle the model could be adapted further around these issues, but more work is needed on arriving at consensus requirements.

The omission of free-text information from various algorithmic approaches to VA interpretation has hindered their acceptance and caused concern over validity[19,20]. One study showed that the sensitivity of VA using physician review for neonatal causes of death was lower when only closed questions were used[29]. Whilst InterVA-M can be used with any kind of VA data, the process of identifying and extracting indicators from open-text often requires greater medical knowledge and subjectivity. Nevertheless, this study suggests little or no benefit from this process, in concordance with an earlier study[30]. It may be the case that free-text information is more informative to physician reviewers than to modelling processes[31], but frequently much information is duplicated between open and closed sections of VA interviews, possibly prolonging the process unnecessarily. Further investigation into the value of free-text information for the InterVA method is anticipated using existing VA data.

The possible precision of VA methods continues to be debated. For public health monitoring, the greatest precision is needed to distinguish between causes that might be the targets of viable interventions. Generating possible multiple causes of death is likely to more accurately reflect the interactions between different diseases that lead to death and more realistically highlight the dominant morbidity and mortality burdens at the community level. Insisting on single causes of death could distort estimates of overall mortality and potential gains from health interventions[19]. Weighting single deaths among several causes could complicate analysis and comparison with other studies[20,32]. However, previous work with InterVA data has incorporated this approach successfully[12,13]. InterVA-M may also be well suited to public health monitoring using the established standards of the international death certificate (ICD-10), which allows for multiple causes in a causal pathway leading to death, however further consideration of interpreting the sequencing of events in InterVA-M is needed.

The InterVA-M model will now move on to being reviewed by a further expert panel, drawn from a range of diverse settings, to review the indicators, possible causes and associated probabilities currently used in the model, together with conceptual and contextual issues regarding terminology and regional variation. A similar process for the all-cause model resulted in an improvement in its overall performance[13]. To ensure that InterVA-M becomes an acceptable tool for use across the developing world, both in research and service settings, we hope to find opportunities for more robust validation studies. A pilot version of the InterVA-M model implemented on a handheld computer (PDA) which allows direct capture and interpretation of VA data is also under test. Meanwhile, the preliminary version of the model, as described here, can be downloaded from the InterVA website[11].

## Conclusion

InterVA-M represents a potentially valuable new tool for objectively measuring maternal mortality and addressing some of the weaknesses of VA methodologies. With further refinement and validation it could become a routine tool for use both in research and service settings where levels and changes in pregnancy-related deaths need to be measured, for example in assessing progress towards MDG-5.

## Competing interests

The author(s) declare that they have no competing interests.

## Authors' contributions

EF – data analysis, model testing and development, drafting and revision

PB – conceiving, developing and evaluating the InterVA-M model

TWO, CT, AG – acquiring and interpreting data from Burkina Faso

IS – data analysis and interpretation

UH – expert clinical reviews of individual cases

KHW, SB – interpretation and model building

TD – acquiring and interpreting data from Ethiopia

SD, JT – acquiring and interpreting data from Ghana

AEF – interpreting data from Bangladesh

NM – interpretation and revision

WJG – interpretation, drafting and revision
